# HIV and Childhood Disability: A Case-Controlled Study at a Paediatric Antiretroviral Therapy Centre in Lilongwe, Malawi

**DOI:** 10.1371/journal.pone.0084024

**Published:** 2013-12-31

**Authors:** Akash Devendra, Atupele Makawa, Peter N. Kazembe, Nancy R. Calles, Hannah Kuper

**Affiliations:** 1 Baylor College of Medicine Children’s Clinical Centre of Excellence (BCOE), Lilongwe, Malawi; 2 Baylor College of Medicine & Texas Children’s Hospital International Paediatric AIDS Initiative (BIPAI), Houston, United States of America; 3 International Centre for Evidence in Disability, London School of Hygiene and Tropical Medicine (LSHTM), London, United Kingdom; University of North Carolina School of Medicine, United States of America

## Abstract

**Background:**

As paediatric antiretroviral therapy (ART) is rapidly scaled up in Southern Africa, Human Immunodeficiency Virus (HIV) infection is becoming a chronic illness. Children growing up with HIV may begin to encounter disabilities. The relationship between HIV, disability and the need for rehabilitation has added an additional element that needs to be addressed by paediatric HIV treatment programmes.

**Study Objectives:**

1) Estimate the prevalence of disabilities in HIV-infected and HIV-uninfected children in Lilongwe, Malawi. 2) Examine types of disability and associated clinical and socio-demographic factors. 3) Identify needs, opportunities and barriers for rehabilitation in Malawi.

**Methods:**

A case-controlled study of 296 HIV-infected children aged 2–9 years attending an ART centre in Lilongwe (cases) and their uninfected siblings (controls) was conducted. Disability was assessed using the WHO Ten Question Screen (TQS). Socio-demographic and clinical data were collected using a parent-proxy questionnaire and medical records.

**Results:**

Of 296 case and control pairs recruited, 33% (98) versus 7% (20) screened positive for a disability (OR 8.4, 4.4–15.7) respectively. Of these 98 HIV-infected cases, 6%, 36%, 33%, 53%, 46% and 6% had a vision, hearing; physical, learning/comprehension, speech or seizure-related disability respectively and 51% had multiple coexisting disabilities. HIV-infected cases with a disability were more likely to be WHO stage III or IV at enrolment (71% vs. 52%, OR 2.7, 1.5–4.2), to have had TB (58% vs. 39%, OR 2.3, 1.4–3.8) and to have below-average school grades (18% vs. 2%, OR 11.1, 2.2–54.6) than those without. Sixty-seven percent of cases with a disability had never attended any rehabilitative service. Twenty-nine percent of caregivers reported facing stigma and discrimination because of the child’s disability.

**Conclusion:**

This study reveals the magnitude of disability among HIV-infected children and the large unmet need for rehabilitation services. This expanding issue demands further investigation to provide an evidence base for holistic care for disabled children living with HIV.

## Introduction

Though still lagging behind adult services, paediatric ART access has undergone rapid expansion across Southern Africa resulting in the life expectancy of children living with HIV increasing substantially; it is now commonplace for perinatally-infected children on ART to live well into late adolescence and adulthood [1]–[3]. In countries such as Malawi, HIV is on the way to being considered a chronic illness [4], [5]. With the shift toward chronicity, children living with HIV may begin to encounter an array of impairments, activity limitations and participation restrictions that can be broadly defined as disabilities [4], [6]–[9]. Disabilities can result from HIV itself, its associated conditions or from medication side effects [6], [10].

High prevalence of disability in people living with HIV has been reported in the West [11]–[14], and more recent studies in low-income countries add to the growing level of knowledge on the extent of these disabilities in resource-limited settings [15]–[18]. However, studies have focussed on adults. To date, there is a lack of prevalence data on disability in children living with HIV in Africa.

Rehabilitation in the context of the HIV epidemic has also been recently receiving overdue attention [19]–[21], though the evidence base remains sparse. Rehabilitation services target the array of needs of people with disability and are thus usually based on a multidisciplinary approach that include physical, occupational or speech-language therapy, mental health interventions and access to assistive devices (e.g. walking aids, prosthetics, orthotics, hearing aids). Longer term interventions may also be offered and these include life skills, educational and vocational support [22]. For resource-limited setting such as Southern Africa where ART delivery is now widespread, there has been a call to understand HIV not only as a medical issue, but also as a disability and rehabilitation concern [6], [8], [21]–[25].

The aims of this study were to:

- Estimate the prevalence of disabilities in HIV-infected children aged 2–9 years in Lilongwe, Malawi (cases), and HIV-uninfected siblings from the same households (controls).
- Examine key associated clinical parameters and sociodemographic factors of HIV-related disability.
- Provide insight into the needs and opportunities of HIV-infected children living with disabilities in Malawi, including rehabilitation or special education services.
- Identify barriers, challenges and stigmatisation that HIV-infected children with disabilities and their caregivers may face.

## Methods

### Ethics Statement

The study protocol was reviewed and approved by both the Baylor College of Medicine Institutional Review Board and the Malawi Ministry of Health National Health Sciences Research Committee. Full written informed consent was obtained and participation was entirely voluntary and confidential. Written informed consent was obtained from the adult caregivers on behalf of the child participants. Participants were free to withdraw at any time without any impact on clinical care.

### Study Design and Setting

This was a case-controlled survey, conducted March to May 2012. Cases were HIV-infected children aged 2–9 years attending for HIV care and treatment services at the Baylor College of Medicine Children’s Clinical Centre of Excellence (BCOE), Lilongwe, Malawi who were matched to HIV-uninfected siblings aged 2–9 living in the same household (controls). In Malawi, the adult HIV prevalence is 12% and there are approximately 120,000 children living with HIV. Of these, nearly 36,000 children are currently receiving ART representing approximately 38% of those eligible [26]–[27]. The BCOE in Lilongwe serves as a tertiary referral centre for paediatric HIV care and treatment and has approximately 3000 active patients enrolled of whom approximately 2200 are on ART [28].

### Participants

Cases were selected by systematic random sampling from among HIV-infected children aged 2–9 years attending the BCOE who were accompanied by an adult caregiver. A case was eligible if he/she had an HIV-uninfected sibling (control), living in the same household and within the same age group (i.e. 2–9 years). Siblings were ineligible as controls if their HIV status was unknown. If the index case had more than one HIV-uninfected sibling, the one closest in age (either older or younger) was chosen. Participants were restricted to the 2–9 year age group to allow the valid use of the WHO Ten Question Screen for Disability [29].

### Data Collection and Tools

Caregivers were interviewed by a trained study assistant about both the case and control subject. Sociodemographic data were collected on both the case and control (age, gender, education, and medical history), as well as information on family members (vital status, education, HIV status) and household (size, location, income, source of water, asset ownership, food security).

The WHO TQS was then administered to the caregiver for both case and control. This internationally validated tool comprises ten questions to assess the presence of disabilities, including delayed developmental milestones, difficulties with sight, hearing, learning/comprehension, movements, speech or seizures [29]–[32]. A disability-positive response to any one of these ten questions indicates a positive screen for disability and a Follow-Up Questionnaire was then conducted with in-depth questions concerning the nature of the disability (severity, history, rehabilitation services, assistive devices, educational support and perceived barriers and challenges).

A parent-proxy Paediatric Quality of Life Inventory (PedsQL) was also administered for both case and control to give quantitative data regarding quality of life [33]. Separate questionnaires were used for the children 2–4 and those 5–7. The questionnaire includes items on physical functioning (8 questions), emotional functioning (5 questions), social functioning (5 questions) and school functioning (5 questions for those 5–7, and 3 questions for those 2–4). Each question was rated on a five point scale from 0 (never a problem) to 4 (almost always a problem).

Finally, clinical information was extracted from the electronic medical records at BCOE for the HIV-infected children (timing of ART, WHO stage, CD4 counts, history of TB, nutritional status, documented disabilities, underlying causes and referrals).

### Data Analysis

Age- and sex-adjusted odds ratios were generated through conditional logistic regression, matching by caregiver, to assess the relationship between HIV case status and the presence of disability. Among the cases, logistic regression analyses were run to assess the association between the presence of disability and, in turn, the HIV characteristics and sociodemographic characteristics of the case. The PedsQL items were reverse-scored and linearly transformed to a 0–100 scale (0–100. 1 = 75, 2 = 50, 3 = 25, 4 = 0) with higher scores indicating better quality of life. Scale scores were computed as the sum of the items divided by the number of items answered. If more than half of the items were missing then the scale score was not calculated. Age-sex-adjusted linear regression analyses were then generated to compare two groups in terms of PedsQL scores (HIV versus no HIV, disability versus no disability), and ANOVA was used for comparison of PedsQL scores in multiple groups (HIV with or without disability).

## Results

### Baseline Characteristics (Table 1)

A total of 296 HIV-infected children (cases) and their uninfected siblings (controls) were recruited. Cases were significantly younger than controls (mean age 5.6 and 6.1 years respectively, p = 0.01) and comprised a similar proportion of males and females (48% and 52% respectively, p = 0.32). The majority (88%) of interviewed caregivers were the HIV-infected child’s mother, 6% the father, 6% aunt or uncle, 3% grandparent, 1% sibling and 1% reported “other” (e.g. neighbour).

**Table 1:** Baseline characteristics of HIV-infected cases and HIV-uninfected controls.

	HIV-Infected Cases(n = 296)	HIV-UninfectedControls (n = 296)	Age-sex adjusted OR (95% CL)
**Age in years (%)**			
**2–3**	29	26	Baseline
**4–5**	30	20	1.2 (0.8–1.8)
**6–7**	25	28	0.9 (0.6–1.2)
**8–9**	17	26	0.6 (0.4–1.0)
**Females (%)**	48	52	0.9 (0.6–1.2)
**Orphan Status (%)**			
Single	16	13	12.4 (1.6–98.8)
Double	1	1	1.3 (0.1–22.1)
**Place of birth** *			
Hospital	50	55	0.8 (0.4–1.7)
Clinic	37	33	1.2 (0.6–2.6)
Home	12	11	Baseline
**Schooling for children >5 years**			
“Always” attends school (%)	42	88	0.2 (0.1–0.4)
Correct school standard for age (%)	49	69	0.2 (0.1–0.7)
“Above-average” school grades (%)	45	62	0.4 (0.2–0.8)
**Medical History**			
Overnight Hospital Admissions (%)	85	32	14.3 (7.9–25.8)
Previous diagnosis of Tuberculosis (%)	45	1	158 (22–1000)
Previous diagnosis of Malnutrition (%)	52	4	151 (21–999)

Not known for 3 subjects.

Children with HIV were significantly less likely to “always” attend school, to be in the correct school standard for age, and to achieve “above-average” school grades compared to their uninfected sibling. In terms of medical history, children with HIV were more likely to have experienced overnight hospital admission and previous diagnosis of TB or malnutrition than their sibling, but there was no difference in place of birth.

### Prevalence of Disability by WHO Ten Question Screen (Table 2) and Types of Disability (Figure 1)

Among the HIV-infected cases, 33% screened positive for a disability compared to 7% of controls (OR 8.4, 95% CI 4.4–15.7). Prior to administration of the TQS, caregivers considered disability to exist for only 10% of cases and 2% of controls (OR 5.8, 2.2–15.0). All the children considered disabled by their caregivers screened positive with the TQS. Seventy percent who screened positive with the TQS were not considered to have a disability by the caregiver.

**Table 2:** Reported disability by WHO Ten Question Screen in HIV-infected cases and HIV-uninfected controls.

WHO Ten Question Screen (TQS)	RelatedDisability	Disability-Positive Response		Age-sex-adjustedOR (95% CL)
		HIV-infected Cases	HIV-uninfected Controls	
1. Does your child have any serious delay in sitting,standing or walking?	Physical	25 (8%)	5 (2%)	4.5 (1.7–12.0)
2. Does your child have difficulty seeing either in thedaytime or at night?	Vision	6 (2%)	0	-
3. Does your child appear to have difficulty hearing?	Hearing	36 (12%)	7 (2%)	6.2 (2.7–14.3)
4. When you tell your child to do something does he/sheseem to understand what you are saying?	Learning	26 (9%)	6 (2%)	4.9 (2.0–12.3)
5. Does your child have difficulty walking or using arms ordoes he/she have weakness or stiffness in the arms/legs?	Physical	30 (10%)	6 (2%)	4.7 (1.9–11.4)
6. Does your child sometimes have fits, become rigidor lose consciousness?	Seizures	6 (2%)	3 (1%)	1.9 (0.5–7.8)
7. Does your child learn to do things like other childrenhis/her age?	Learning	37 (13%)	7 (2%)	7.5 (2.9–19.3)
8. Does your child speak at all?	Speech	21 (7%)	2 (1%)	9.7 (2.3–41.9)
9a. For 3–9 year olds: Is your child’s speech any waydifferent from normal?	Speech	38 (15%)	3 (1%)	10.0 (3.0–32.8)
9b. For 2 year olds: Can your child name at leastone object?	Speech	8 (24%)	1 (3%)	11.5 (1.3–98.5)
10. Compared with other children his/her age doesyour child appear in any way mentally backward, dullor slow?	Learning	51 (17%)	6 (2%)	12.5(4.5–34.9)
Parent reported disability		31 (10%)	5 (2%)	5.8 (2.2–15.0)
**Positive Screen for Disability**		**98 (33%)**	**20 (7%)**	**8.3(4.4–15.7)**

**Figure 1:**
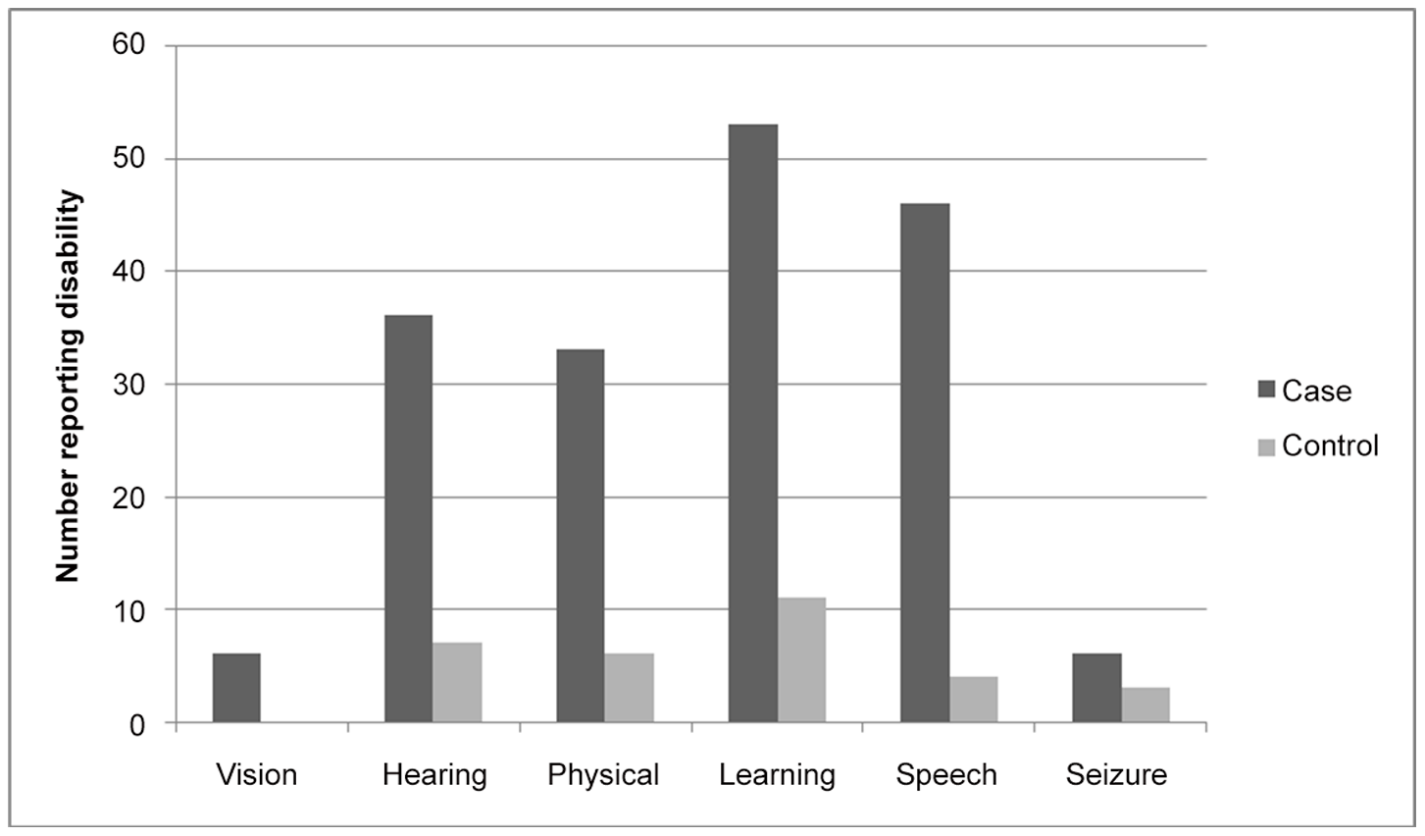
Types of disability by HIV status.

Based on the TQS, types of disability were categorised into the 6 following groups: vision, hearing, physical, learning/comprehension, speech or seizure-related disability. A total of 180 different disabilities were reported among the 98 children. Of the 98 HIV-infected children screening positive for disability, 49% had a single disability, while 29% had 2 co-existing types of disability and 22% had 3 or more different co-existing types of disability. Similarly, among the 20 HIV-uninfected children with disability, 55% had a single disability and 35% had 2 co-existing types, but only 10% had 3 or more types. Figure 1 demonstrates the distribution of these different disabilities. For all types of disability, excepting seizures, there was a large and significantly positive response in the HIV-infected group in comparison to the controls.

### The Paediatric Quality of Life Inventory (Table 3)

The PedsQL Inventory Scores were compared among children with and without HIV, with and without disability, and with and without HIV and disability (Table 3). Children with HIV scored significantly worse than children without HIV in terms of total functioning, physical health, and emotional, social and school functioning. Similarly, children with disability scored significantly worse than children without disability in all of those domains. When comparing functional status in children by both HIV and disability status, it was apparent that those children with both disability and HIV had the poorest scores, followed by children with disability but no HIV and then children with HIV but no disability. The largest differences between the groups was in the domain of school functioning.

**Table 3:** PedsQL Inventory Scores comparing children by HIV status and presence of disability.

	Total Score*	Physical Health*	Emotional functioning*	Social functioning*	School functioning* ^+^
**HIV**					
No HIV (n = 296)	87.1 (8.6)	85.9 (11.5)	86.6 (12.3)	90.4 (13.5)	87.2 (12.7)
HIV-infected (n = 294)	79.1 (12.2)	77.9 (15.4)	80.9 (12.9)	82.4 (18.5)	72.9 (18.5)
P-value**	<0.0001	<0.0001	<0.0001	<0.0001	0.0004
**Disability**					
No disability (n = 472)	86.1 (7.9)	84.8 (9.2)	85.7 (11.8)	89.6 (13.2)	85.4 (12.6)
Disability (n = 118)	71.3 (14.8)	70.2 (22.2)	76.1 (14.2)	73.5 (22.3)	63.7 (20.8)
P-value**	<0.0001	<0.0001	<0.0001	<0.0001	<0.0001
**HIV and disability**					
No HIV/no disability (n = 276)	88.1 (7.1)	87.0 (8.9)	87.2 (11.8)	91.5 (12.0)	88.4 (11.7)
HIV/no disability (n = 196)	83.3 (8.0)	81.9 (8.7)	83.5 (11.5)	87.0 (14.3)	79.7 (12.2)
No HIV/disability (n = 20)	74.0 (15.2)	71.1 (25.7)	78.5 (15.9)	75.0 (22.4)	74.1 (15.0)
HIV/disability (n = 98)	70.8 (14.8)	70.0 (21.6)	75.6 (13.9)	73.2 (22.4)	60.1 (21.4)
P-value***	<0.0001	<0.0001	<0.0001	<0.0001	<0.0001

Higher scores relate to better quality of life.

Age and sex adjusted p-value.

P-value from analysis of variance.

Data on school functioning were available for 167 children with no HIV and no disability, 16 children with no HIV and disability, 89 children with HIV and no disability, and 47 children with HIV and disability.

### Clinical Characteristics of HIV-infected Cases in Relation to Disability (Table 4)

Treatment with ART and duration of treatment were not associated with the presence of disability among children with HIV, nor were there differences in CD4 levels between children with or without disability. Cases with disability were more likely to be WHO stage III or IV at enrolment than cases without disability, and were more likely to have had previous treatment for TB. There was no significant difference in the prevalence of reported malnutrition for children with or without disability. There were no clear variations in these associations when assessed separately for younger (<5 years) and older (> = 5 years) children (data not shown).

**Table 4:** Clinical characteristics of HIV-infected children in relation to disability.

Clinical Characteristics	Total group		
	HIV-infected with disability(n = 98)	HIV-infected without disability (n = 198)	Age and sex adjusted OR(95% CL)
On ART:			
Yes	91 (93%)	175 (89%)	1.8 (0.7–4.5)
No	7 (7%)	22 (11%)	Baseline
Age started ART (if on ARVs):			
<1.5 years	28 (31%)	60 (34%)	Baseline
1.5–3 years	31 (34%)	58 (33%)	1.1 (0.6–2.0)
>3 years	32 (35%)	57 (33%)	0.9 (0.4–1.9)
WHO stage at enrolment			
I	17 (17%)	64 (32%)	Baseline
II	11 (11%)	31 (16%)	1.2 (0.5–3.1)
III	52 (53%)	84 (42%)	2.5 (1.3–4.8)
IV	18 (18%)	19 (10%)	3.8 (1.6–9.0)
Overnight hospital admission	90 (92%)	161 (81%)	2.7 (1.2–6.2)
Past diagnosis of TB	57 (58%)	77 (39%)	2.3 (1.4–3.8)
Past diagnosis of malnutrition	54 (55%)	100 (51%)	1.3 (0.8–2.1)
Absolute CD4 at enrolment	775 (601)	870 (602)	P = 0.23
CD4% at enrolment	19 (10)	20 (10)	P = 0.94

### Socio-demographic Factors in HIV-infected Cases in Relation to Disability (Table 5)

There were few clear sociodemographic correlates of disability among children with HIV. Factors including orphanhood, asset ownership, rurality, family income or parents’ education were not found to be associated with disability among children with HIV. In children above 5 years, those with disability were more likely to have below-average grades compared to those without disability. In addition, there was some evidence that lack of sufficient food in the household was related to the presence of disability.

**Table 5:** Sociodemographic characteristics of HIV-infected children with relation to disability.

		HIV-infected with disability	HIV-infected withoutdisability	Age- and sex- adjusted OR (95% CL)
	**Single or double orphan**	17 (17%)	34 (17%)	1.0 (0.5–1.9)
	**Number living in household:**			
	<4	31 (32%)	58 (29%)	Baseline
	4–6	42 (43%)	90 (45%)	0.8 (0.5–1.5)
	>6	25 (26%)	50 (25%)	0.9 (0.5–1.8)
	**Asset ownership:**			
	0	24 (24%)	65 (33%)	Baseline
	1	51 (52%)	85 (43%)	1.7 (0.9–3.0)
	2–3	10 (10%)	28 (14%)	1.0 (0.4–2.4)
	4–5	13 (13%)	20 (10%)	1.8 (0.8–4.2)
**Household**	**Time taken to reach clinic:**			
**Characteristics**	<60 minutes	32 (33%)	77 (39%)	Baseline
	1–2 hours	49 (50%)	93 (47%)	1.3 (0.8–2.3)
	>2 hours	17 (17%)	28 (14%)	1.5 (0.7–3.1)
	**Sufficient food in household:**			
	-Always enough	13 (13%)	44 (22%)	Baseline
	-Sometimes not enough	46 (47%)	89 (45%)	1.8 (0.9–3.6)
	-Often not enough	21 (21%)	38 (19%)	1.9 (0.8–4.4)
	-Never enough/poor quality	18 (18%)	26 (13%)	2.5 (1.0–6.0)
	**Family combined income:**			
	<$52 per month	54 (57%)	127 (66%)	Baseline
	$52–$187 per month	37 (39%)	53 (28%)	1.6 (0.9–2.7)
	>$187 per month	3 (3%)	22 (6%)	0.6 (0.1–2.1)
	**Location:**			
	-City	8 (8%)	17 (9%)	1.1 (0.4–3.0)
	-Peri-urban	73 (74%)	144 (73%)	1.2 (0.6–2.2)
	-Rural	17 (17%)	36 (18%)	Baseline
	**Highest education of mother:**			
	-No formal schooling	8 (8%)	18 (%)	0.8 (0.3–2.0)
	-Primary	45 (46%)	101 (51%)	0.8 (0.5–1.4)
**Parental**	-Secondary or higher	45 (46%)	79 (40%)	Baseline
**Characteristics**	**Highest education of father:**			
	-No formal schooling	5 (5%)	9 (5%)	1.0 (0.3–3.1)
	-Primary	30 (31%)	78 (39%)	0.7 (0.4–1.1)
	-Secondary or higher	63 (64%)	111 (56%)	Baseline
	**Child attending school:**			
	-No	13 (22%)	12 (12%)	1.9 (0.7–5.1)
	-Sometimes	22 (37%)	48 (47%)	0.7 (0.4–1.5)
	-Always	25 (42%)	43 (42%)	Baseline
**Schooling**	**Child in standard correct for age:**			
**(Age >5 years**	-No	26 (55%)	43 (48%)	1.6 (0.7–3.5)
**only)**	-Yes	21 (45%)	46 (52%)	Baseline
	**Child’s grades:**			
	-Below average	11 (18%)	2 (2%)	11.1 (2.2–54.6)
	-Average	19 (32%)	44 (43%)	0.9 (0.4–1.8)
	-Above average	17 (28%)	45 (44%)	Baseline

### Healthcare and Rehabilitative Services (Table 6)

The majority (67%) of the 98 cases with HIV who screened positive with the TQS reported never having attended any rehabilitative assessment or service for a disability. One third (34%) of caregivers reported never having discussed their disability concern with a health professional.

Table 6 shows the range of services attended by HIV-infected disabled children and also demonstrates the unmet needs for specific types of services. For example only 14% of children reporting hearing difficulties had been seen in Ear Clinic, only 57% with reported visual impairments seen in Eye Clinic and only 42% with physical issues had ever attended Physiotherapy.

**Table 6:** Met and unmet rehabilitation needs for HIV-infected children with disabilities.

Facility/service attended for disability	Number of HIV-infectedcases who have attended	% of the total number of cases with the type of disability that would benefit from this service, who have attended this service
Hospital	29	n/a
General Outpatient Clinic	9	n/a
Specialist Clinic (with referral)	21	21%
Eye Clinic	4	57%
Ear Clinic	5	14%
Multidisciplinary Disability Clinic	12	12%
Seizure Clinic	0	0%
Physiotherapy	15	42%
Private Doctor	1	n/a
Traditional Healer	1	n/a
Special Needs School	5	n/a
Other	1	n/a

Of the children who had attended services, 41% of caregivers reported these being helpful, while half reported these as not helpful (24%) or partially helpful (35%). The majority (69%) of respondents believed specialist clinics could help their child (15% ear clinics, 10% speech therapy clinics, 6% eye clinics, 5% orthopaedic clinics and 64% multidisciplinary clinics), while one third (35%) of caregivers believed there were particular items of equipment that could be helpful for their child with disabilities (including hearing aids (n = 16), wheelchairs (n = 2), bicycles (n = 3), crutches or walking aids (n = 6), glasses (n = 2) ). Only 5% of children with HIV and disability had ever received any rehabilitative equipment in the past and only 1% of respondents had ever received any extra income or disability support from the government or non-governmental organisations because of the child’s disability. Just 8% of these caregivers had ever attended a disability support group.

The main reported barrier to accessing disability-related services was lack of money for transport (60%), followed by services too far (20%), and lack of funds for services/equipment (16%). Nine percent of caregivers reported at least one episode of healthcare professionals being unhelpful, discriminatory, not listening or lacking in sufficient training with respect to their child’s disability. Twenty-nine percent of caregivers report facing stigma or discrimination in the community, at school or in the healthcare sector because of the child’s disability.

### Electronic Medical Records Review

Of the 98 cases with HIV who had a positive TQS for disability, only 36% had any disability or special needs ever documented in their medical record at the BCOE and only 17% had any documentation or reference to their special needs or disability in the last 3 consultations.

The distribution by type of disability in the 36 cases was as follows: vision (n = 4), hearing (n = 8), physical (n = 7), learning/comprehension (n = 4), speech (n = 5), seizures (n = 1) and global developmental delay (n = 12).

The underlying causes reported in the electronic records included HIV encephalopathy (n = 12), vasculitis (n = 1), tuberculous meningitis (n = 5), malaria (n = 4), cerebral palsy (n = 1), rickets (n = 1), severe malnutrition (n = 2), bacterial meningitis (n = 2), head injury (n = 1) and chronic suppurative otitis media (n = 3). 4 cases had no underlying diagnosis documented. Among those with documented disability, only 23% had a confirmed referral recorded with regards to the disability.

## Discussion

The findings of this case-controlled study demonstrated that one third of children with HIV had a disability, and that the odds of disability were more than eight times higher in the HIV-infected child compared to his/her uninfected sibling. The prevalence of disability among the HIV-uninfected control population is comparable to estimates reported in other areas of Southern Africa [34].

Prior to administration of the screening test, caregivers considered a disability to exist for only 10% of children with HIV and 2% of HIV-uninfected siblings, so that 70% of cases were under-reported. This may highlight the pervasive lack of understanding amongst lay people of what defines a disability. That over a third of HIV-infected children who screened positive for a disability had not ever had any impairment brought to the attention of a health professional may also indicate low levels of awareness amongst caregivers surrounding disabilities and rehabilitation.

Disabilities among HIV-infected children were commonly not found in isolation; approximately half of all cases had two or more co-existing disabilities. The disabilities that were noted occurred across a broad spectrum. Of cases who tested positive for a disability approximately half had a learning-related problem, half had a speech-related difficulty, a third a hearing impairment and a third had a physical impairment. These figures can provide some indication to the requirement for various rehabilitative services such as hearing clinics, physiotherapy, or speech therapy. However, given that these disabilities frequently co-exist, it seems imperative that services should be multidisciplinary and holistic.

HIV and many of its associated conditions such as malnutrition and TB have multi-system effects and a propensity to affect the developing brain in growing children. It is thus no surprise that there exists a pattern for multiple co-existing disabilities and delayed developmental milestones. Likewise, the significantly increased risk for screening positive for a disability with late presentation of HIV (WHO stage III or IV) or a previous history of TB adds weight to this.

It was apparent that those children with disability had worse quality of life scores for physical, emotional, social, school and total functioning, compared to children without disability, both among children with HIV and those without HIV. There was an interesting trend for poorer educational outcomes associated with disability with nearly twice the number of HIV-infected and disabled children never attending school and nearly a ten times likelihood of having “below average” grades. These results support the anecdotal experience of clinicians dealing with HIV-infected children who are found to perform poorly in school with resulting parental concerns.

There is increasing evidence regarding HIV-associated neuro-cognitive effects in children, their assessment and their impacts on education, though few studies have been conducted in Southern Africa [35], [36]. It is interesting to note that the educational outcomes in HIV-infected children were significantly worse than their HIV-uninfected siblings, even when they screened negative for disability and that among the children without disability those who were HIV positive had poorer functioning scores compared to those who were HIV negative. This may indicate that the WHO TQS may not have been sensitive enough to include all those children performing poorly at school.

The results pertaining to the healthcare and rehabilitative services demonstrated an overwhelming unmet need. This survey was conducted at a large well-resourced ART clinic on the grounds of the main tertiary referral hospital in Lilongwe. Despite this, two-thirds of cases screening positive for a disability had never attended any rehabilitative service. Very small numbers of patients and caregivers had ever received any specialised disability related equipment, financial support or attended a support group. Reported barriers to accessing rehabilitative services were similar to those to accessing healthcare services in general in Malawi and include distance and transport costs. Important findings which deserve attention are the perceived discrimination from healthcare workers and the lack of awareness from caregivers as to what rehabilitation itself entails. That nearly one third of caregivers faced discrimination or stigma in the community, schools or healthcare sector because of the disability is a great concern, given the already high levels of stigma surrounding HIV itself [37].

Lastly, the results from the medical records review highlighted a strong trend for clinicians to focus on the biomedical aspects of HIV care whilst paying little attention to holistic issues such as disability. Lack of awareness, inadequate training and poor knowledge of local disability services and referral pathways may all contribute. Comprehensive mapping of disability-focused services in Malawi would be essential.

The most important limitation to consider in this study was the lack of definitive clinical confirmation of impairment after a positive WHO TQS screen. The TQS provided a very simple, quick and cheap way to screen for disabilities and has been internationally validated, including within Southern Africa [29], [30]. Although it has been found to have a low positive predictive value for serious disability, ‘false positive’ cases are often found to have a mild disability and thus the results from this study are useful in providing an inclusive picture [29], [30]. Ideally however, the TQS should be part of a two-step process wherein children with a positive screen are referred for further clinical evaluation or treatment [29]. This was outside the scope of this study.

Measuring disability in itself is notoriously challenging [29]. The World Report on Disability advocates for the adoption of the WHO’s International Classification of Functioning, Disability and Health (ICF) as the standard framework for disability data collection whilst others have developed more HIV-specific tools such as the HIV Disability Questionnaire (HDQ) [38], [39]. Most tools however are lengthy and difficult to administer.

The numbers included in this study were not large enough to provide significance for some trends such as educational outcomes and seizure-related disabilities. As the population surveyed was attending a large urban tertiary referral centre, it may not be entirely representative of all children with HIV in Malawi, and similar studies at semi-urban or rural clinics and district general hospitals will provide a useful comparison.

This is the first study we know of providing data on the prevalence of disability in children living with HIV in Africa. The design incorporating a matched HIV-negative control group offers important comparison data, and using the same respondent for both cases and controls would reduce responder bias. The use of a sibling as control would reduce the variation between the cases and controls to being large in terms of factors related to HIV infection, rather than socio-demographic differences. The results give valuable insight into the magnitude of the problem, the large unmet need for assessment and rehabilitation services and the challenges faced by disabled HIV-infected children and their caregivers. Though this study focused on children, these issues are also highly applicable to adults living with HIV. Malawi is a resource-limited, high HIV prevalence country with stretched healthcare services that are comparable to those in many countries in the region and this data should open up thinking about longer term challenges in the era of increasing access to paediatric ART in Africa.

## Conclusions

A high magnitude of disability was demonstrated in HIV-infected children and access to rehabilitation services found to be lacking. Disability will affect many children and adults living with HIV in the years ahead and HIV programmes must respond to these evolving needs. This expanding but neglected field demands fuller evaluation to provide an evidence base for holistic care at the community, clinical and policy levels.
